# Prevention of Noise-Induced Hearing Loss In Vivo: Continuous Application of Insulin-like Growth Factor 1 and Its Effect on Inner Ear Synapses, Auditory Function and Perilymph Proteins

**DOI:** 10.3390/ijms24010291

**Published:** 2022-12-24

**Authors:** Kathrin Malfeld, Nina Armbrecht, Andreas Pich, Holger A. Volk, Thomas Lenarz, Verena Scheper

**Affiliations:** 1 Department of Otolaryngology, Hannover Medical School, Carl-Neuberg-Str. 1, 30625 Hannover, Germany; 2 Department of Small Animal Medicine and Surgery, University of Veterinary Medicine Hannover, Foundation, 30559 Hannover, Germany; 3 Core Facility Proteomics, Hannover Medical School, Carl-Neuberg-Str. 1, 30625 Hannover, Germany; 4 Cluster of Excellence “Hearing4all”, German Research Foundation (DFG; “Deutsche Forschungsgemeinschaft”), Hannover Medical School, Carl-Neuberg-Str. 1, 30625 Hannover, Germany

**Keywords:** temporary threshold shift, ribbon synapse, proteomics, synaptopathy

## Abstract

As noise-induced hearing loss (NIHL) is a leading cause of occupational diseases, there is an urgent need for the development of preventive and therapeutic interventions. To avoid user-compliance-based problems occurring with conventional protection devices, the pharmacological prevention is currently in the focus of hearing research. Noise exposure leads to an increase in reactive oxygen species (ROS) in the cochlea. This way antioxidant agents are a promising option for pharmacological interventions. Previous animal studies reported preventive as well as therapeutic effects of Insulin-like growth factor 1 (IGF-1) in the context of NIHL. Unfortunately, in patients the time point of the noise trauma cannot always be predicted, and additive effects may occur. Therefore, continuous prevention seems to be beneficial. The present study aimed to investigate the preventive potential of continuous administration of low concentrations of IGF-1 to the inner ear in an animal model of NIHL. Guinea pigs were unilaterally implanted with an osmotic minipump. One week after surgery they received noise trauma, inducing a temporary threshold shift. Continuous IGF-1 delivery lasted for seven more days. It did not lead to significantly improved hearing thresholds compared to control animals. Quite the contrary, there is a hint for a higher noise susceptibility. Nevertheless, changes in the perilymph proteome indicate a reduced damage and better repair mechanisms through the IGF-1 treatment. Thus, future studies should investigate delivery methods enabling continuous prevention but reducing the risk of an overdosage.

## 1. Introduction

With a global proportion of 16% noise-induced hearing loss (NIHL), a form of sensorineural hearing loss (SNHL), is one of the leading causes of occupational diseases amongst adults. It is caused by either one-time exposure to high level noise or by continuous ambient exposure [1]. Different occupational groups are at risk, such as military pilots [2], firefighters [3] and police officers [4], but also dentists [5], preschool teachers [6] or professional musicians [7,8]. NIHL is not only an occupational disease, but also affects young people. This way 1.1 billion adolescents and young adults are at risk and approximately 5% of the world’s population suffers from NIHL [9,10]. As a form of SNHL it is characterized by a hearing threshold elevation and the loss of cochlear sensory cells. Subsequently, the spiral ganglion neurons (SGN) and their central projections die [11,12,13]. Unfortunately, damage to the cochlear structures can occur even without a visible increase in the hearing threshold. This “hidden hearing loss” (HHL) is caused by a synaptopathy of the ribbon synapses between the inner hair cells and the peripheral dendrites of the SGN [14,15,16,17]. As a result, the wave I amplitude is reduced in the auditory brainstem response (ABR) and patients struggle with hearing in noisy environments [18].

Since up to now there are no reliable treatment strategies for NIHL, prevention is the method of choice—conventionally with earplugs or other hearing protection devices. The outcome of such devices varies dependent on the user’s compliance and in some work situations, e.g., in the kindergarten, the use is impossible. As a concept to circumvent this problem, pharmacological prevention is investigated in several animal and some human studies [19,20,21]. Many studies are exploring the effect of antioxidants as they can potentially neutralize the reactive oxygen species (ROS) that play a key role in the pathogenesis of SNHL and NIHL in particular [22,23,24,25,26,27]. The cochlea is a metabolically active organ and therefore its cells have many mitochondria, especially cells in the stria vascularis. Additionally, under physiological conditions they produce ROS by the electron transport chain. During noise exposure the metabolic activity increases and a reduced cochlear blood flow [28,29,30] leads additionally to oxygen deficiency at the mitochondria. As a result of both processes, the ROS-production increases. Unfortunately, after noise exposure, the reperfusion makes more oxygen available at the mitochondria which also increases the ROS production.

Insulin-like growth factor 1 (IGF-1) is a polypeptide with 70 amino acids and is synthetized in the liver. For IGF-1, antioxidant effects have been described in various disease models such as liver cirrhosis [31,32] or cardiomyopathy [33]. It acts on target cells by binding on its specific receptor, which is also expressed in the cochlea [34]. In vitro IGF-1 induces the regeneration of damaged cochlear ribbon synapses [35] and therefore minimizes cochlear synaptopathy. Animal studies revealed that an intratympanic delivery of IGF-1 via hydrogel or ultrasound microbubbles has preventive and therapeutic effects in NIHL [36,37,38] and ischemic cochlear damage [39]. IGF-1 has also been successfully used in humans to treat sudden sensorineural hearing loss [40,41].

However, regarding NIHL, all up to now published studies have one limitation: they all investigated temporary delivery. Unfortunately, in many situations, the timing of acoustic trauma cannot be accurately predicted, or cumulative effects of noise [42] occur. To cover the time a noise insult damages the inner ear, a drug delivery for pharmacological prevention should take place continuously. The present study aimed to investigate the potential of continuous IGF-1 delivery to prevent noise-induced temporary threshold shift (TTS) and to improve the recovery thereafter. A moderate TTS-inducing noise trauma was applied [43]. As the biggest recovery of the synapses occurs during the first week after noise trauma [44] a recovery phase of seven days was used. This proof-of-concept study might provide the basis for preventive treatment studies of NIHL in the future.

## 2. Results

**Figure 1:**
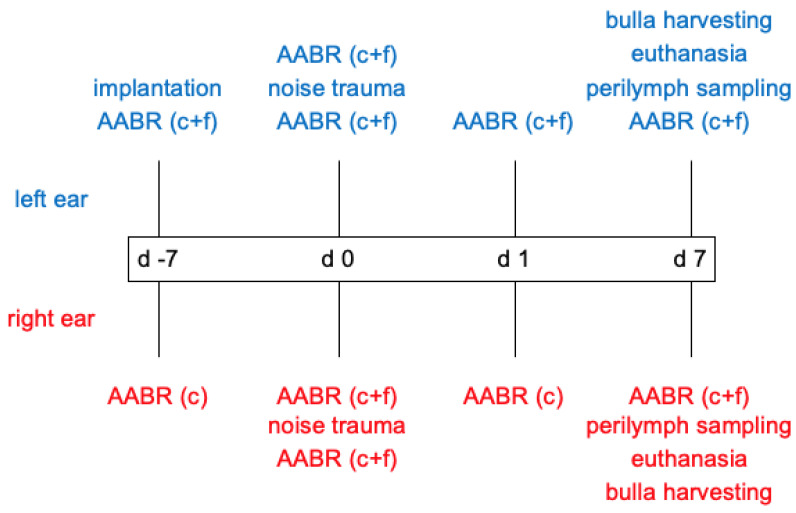
Overview over the experimental timeline, respective interventions, and scope of the AABR measurements for implanted (left, blue) and contralateral (right, red) ears. c = click, f = frequency-specific (0.5 kHz, 1 kHz, 2 kHz, 4 kHz, 8 kHz, 16 kHz, 32 kHz, 40 kHz).

Guinea pigs were used as animal model. One week before noise insult (d -7) the animal’s normal hearing was verified by acoustically evoked auditory brainstem response (AABR) measurement and subsequently an IGF-1-delivering device (hook-delivery device with osmotic pump, see Section 4.4) was implanted in the treatment group (*n* = 7). The control group (*n* = 6) received an osmotic pump containing artificial perilymph (AP). On day 0 an additional AABR was performed followed by 4 h noise exposure and a second AABR measurement 30 min after the noise insult. After 1 day (day 1) an AABR measurement and a µCT to analyze to implant’s position were performed. One week after noise insult, on day 7, a final AABR was followed by µCT imaging, apical perilymph sampling and euthanasia. For a detailed overview over the experimental timeline and performed AABR measurements see Figure 1.

### 2.1. Acoustically Evoked Auditory Brainstem Response (AABR)-Measurements

**Figure 2:**
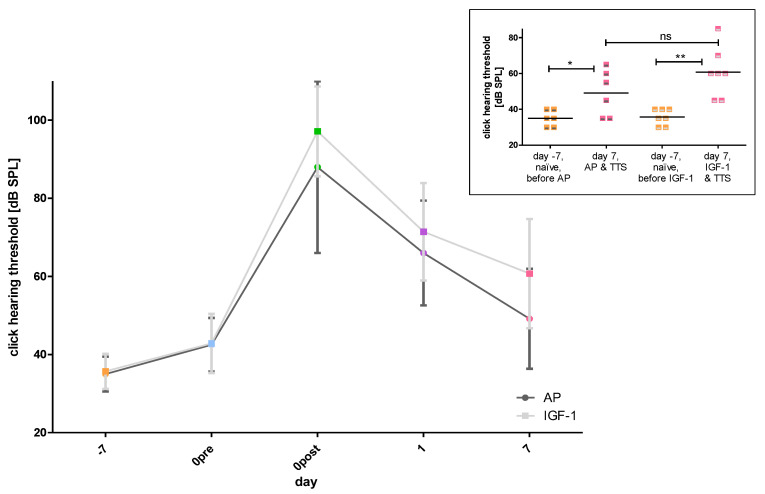
The click hearing thresholds of the implanted ears are illustrated. (**A**) The mean hearing thresholds of the two treatment groups (AP (dark grey) and IGF-1 (light grey)) are displayed over time (day −7 orange, day 0 pre noise blue, day 0 post noise green, day 1 violet, day 7 pink). (**B**) The individual thresholds and the means of both treatment groups, when included into the study (day −7, naïve, orange) and 7 days after TTS (pink) are illustrated. In both groups a significant threshold increase is observed with a larger before-after difference in the IGF implanted ears than in the AP implanted ears. One week after TTS the hearing threshold of the two groups did not differ significantly. ns = not significant; * *p* ≤ 0.05; ** *p* ≤ 0.01.

To get an overview of the influence of implantation and noise trauma as well as possible group differences between implanted ears, the progression of the click hearing threshold over time was considered first (Figure 2A). The initial hearing threshold was the same in both groups and both groups showed a similar shaped curve. Nevertheless, there was a non-significant trend for slightly higher thresholds in IGF-1 treated animals compared to the AP treated ones at day 0 post noise trauma (97.14 ± 11.5 dB SPL versus (vs.) 88 ± 21.97 dB SPL), day 1 (71.43 ± 12.49 dB SPL vs. 66 ± 13.42 dB SPL) and day 7 (60.71 ± 13.97 dB SPL vs. 49.17 ± 12.81 dB SPL). The click evoked hearing thresholds at day 7 did not differ significantly between groups but the click thresholds on day −7 (naïve, before any treatment) and day 7 (i.e., 7 days after noise trauma) differed significantly within groups (Figure 2B).

#### 2.1.1. Impact of the Implantation on Hearing Threshold

**Figure 3:**
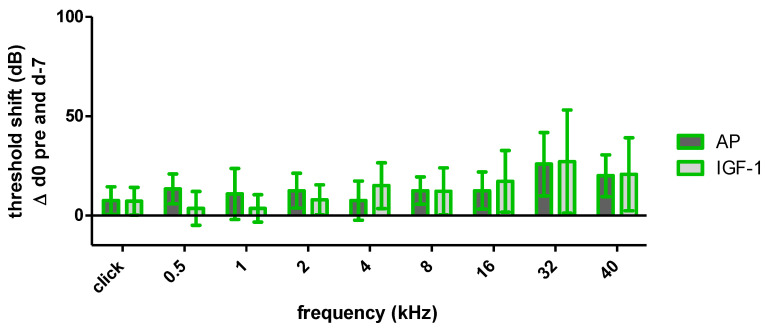
Mean and standard deviation (SD) threshold shift of the implanted ears of both treatment groups one week after surgery before noise trauma. AP displayed in dark grey and IGF-1 in light grey. There was no significant difference between groups.

As the click evoked hearing thresholds on day 0 pre noise did not differ significantly between AP and IGF-1 treated ears, the click and frequency specific threshold shifts between day −7 and day 0 pre noise, were considered in order to provide information about the surgery-related threshold shift (Figure 3). Both groups showed an increase in click evoked and frequency specific thresholds, but the threshold shifts did not differ significantly between groups. In the high frequency region (32 kHz and 40 kHz), where the implant is located, the threshold shift exceeds 20 dB, but in middle and low frequencies the threshold shift is more flattened and below 20 dB. Therefore, both groups have the same initial situation for noise trauma. Nevertheless, the IGF-1 group showed a tendency towards smaller shifts in the low frequencies (0.5, 1 and 2 kHz), whereas at 4 kHz the shift was slightly higher.

#### 2.1.2. Impact of Noise Trauma and Treatment on Hearing Threshold

**Figure 4:**
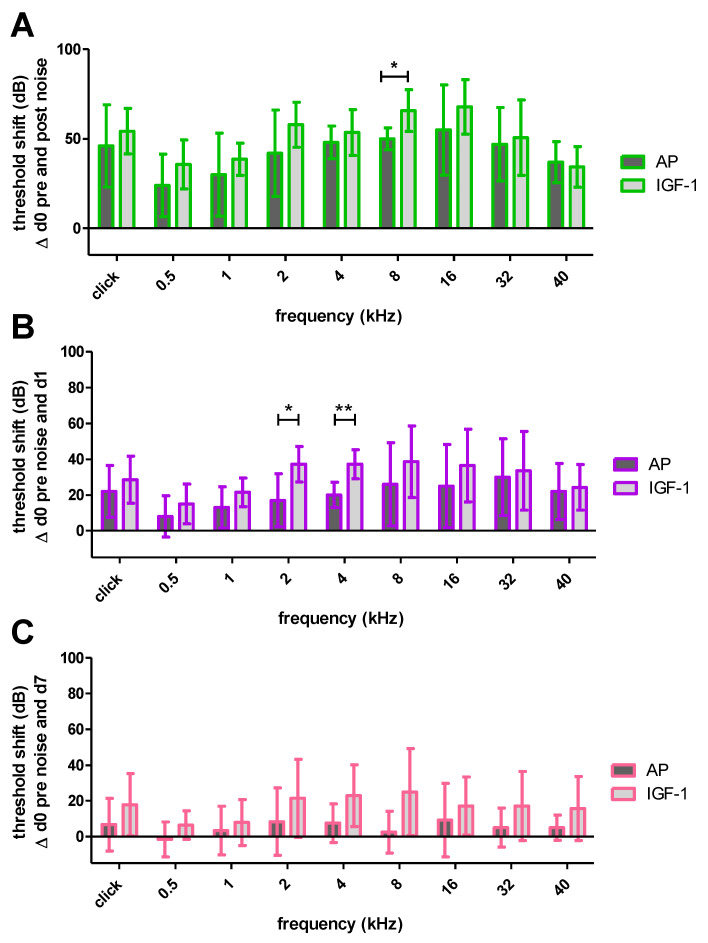
Mean and SD noise-induced threshold shifts of the implanted ears of both treatment groups at different time points. (**A**) 30 min after the noise exposure the IGF-1 group showed a significantly higher threshold shift at 8 kHz. (**B**) One day after the noise exposure there is a frequency specific recovery with significantly higher shifts at 2 and 4 kHz in the IGF-1 treated animals. (**C**) One week after the noise trauma the recovery proceeds and both groups do not show significant differences anymore. Only significant differences are illustrated in A, B and C; * *p* ≤ 0.05; ** *p* ≤ 0.01.

Thirty minutes after the TTS-inducing noise the implanted ears of both groups showed a threshold shift with all frequencies being affected (Figure 4A). Although the IGF-1 treated animals showed a slightly higher shift in all frequencies, only the shift at 8 kHz was significantly increased compared to the AP treated animals (65.71 ± 11.7 dB SPL vs. 50 ± 6.12 dB SPL; *p* ≤ 0.05). One day after noise trauma the hearing threshold in both groups recovered, resulting in a decreased threshold shift (Figure 4B). Still, most of the frequencies did not differ significantly between treatment groups, except for 2 kHz (37.14 ± 9.94 dB SPL vs. 17 ± 14.83 dB SPL; *p* ≤ 0.05) and 4 kHz (37.14 ± 8.09 dB SPL vs. 20 ± 7.07 dB SPL; *p* ≤ 0.01). One week after noise trauma the recovery proceeded and partially no noise-induced threshold shift was detectable anymore (Figure 4C). At this time point there was no significant difference in any tested frequency. However, there was a tendency for larger threshold shifts in IGF-1 treated animals, especially in the middle frequencies 2 kHz (21.43 ± 21.93 dB SPL vs. 8.34 ± 18.89 dB SPL), 4 kHz (22.86 ± 17.29 dB SPL vs. 7.5 ± 10.84 dB SPL) and 8 kHz (25 ± 24.32 dB SPL vs. 2.5 ± 11.73 dB SPL) that failed to reach significance.

#### 2.1.3. Impact of Noise Trauma on Contralateral Ears

**Figure 5:**
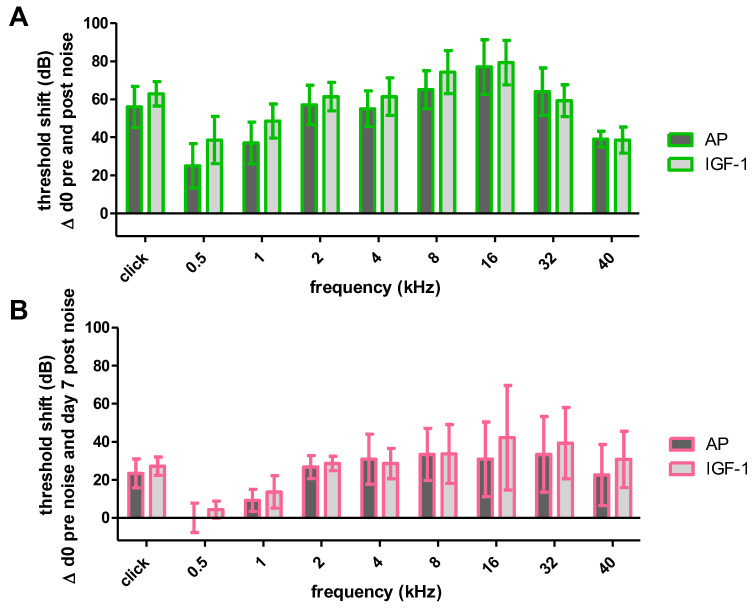
Noise-induced threshold shifts of the contralateral ears of both treatment groups (AP (dark grey) and IGF-1 (light grey). Neither 30 min (**A**) nor one week after noise trauma (**B**) there are significant differences between both groups. Mean and SD are plotted.

In order to examine potential effects of the IGF-1 delivery on the contralateral ears, the noise-induced threshold shifts of the contralateral ears of the IGF-1- and AP-treated animals were compared 30 min (Figure 5A) and 7 days after (Figure 5B) noise trauma. No significant differences between groups were observed at any time point. Only negligible higher threshold shifts were detected in the contralateral ears of IGF-1 animals at day 7 in the higher frequencies 16, 32 and 40 kHz.

### 2.2. Perilymph

**Figure 6:**
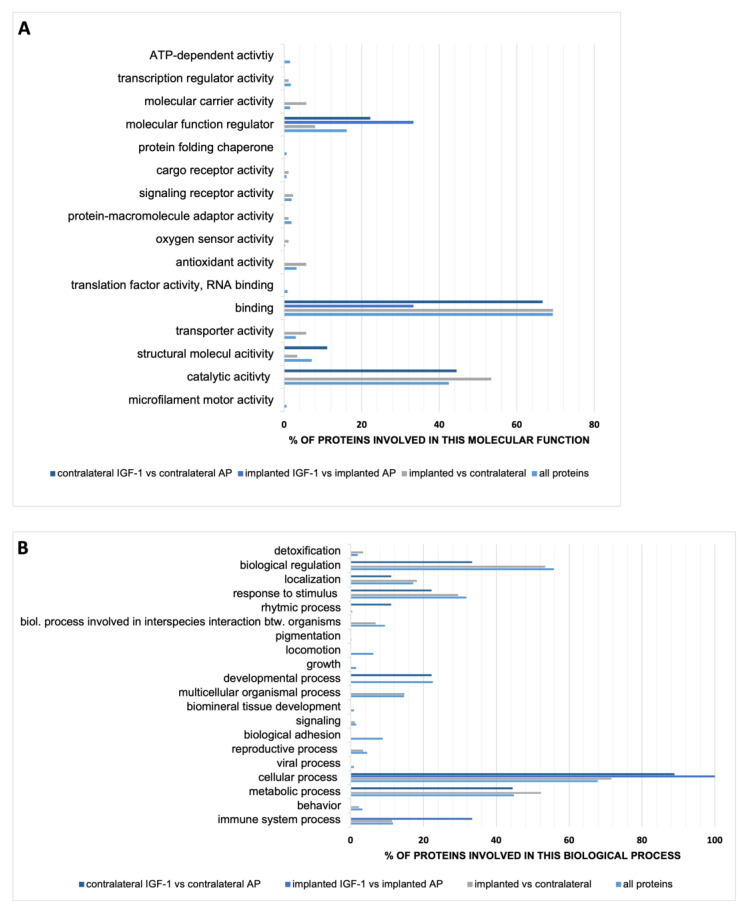
Characterization of guinea pig perilymph proteins by GO profiles by (**A**) molecular function and (**B**) biological process. Categories are counted nonexclusively, so a protein may count in more than one category. The total number of proteins varies per group. All proteins display the 466 quantified proteins. The other groups display only significant varied proteins, which are 88 for the comparison implanted (both IGF-1 and AP) vs. contralateral ears, 3 for the comparison implanted with IGF-1 vs. implanted with AP and 9 for the comparison of the contralateral ears of both treatment group. Accordingly, the counts mapped to the categories molecular function and biological process vary in number and were used as 100% value. Therefore, the counts are standardized and illustrated in %.

The obtained volume varied from 0.5 µL to 2.5 µL, except for one sample that was only one drop (~0.05 µL). Since the total volume of the guinea pig perilymphatic space is ~8.87 µL and ~4.66 µL for the scala tympani [45], we collected maximum a quarter of the whole perilymph volume using capillary forces. All samples underwent LC-MS analysis and a total of 1478 proteins were identified. Out of them 466 proteins were quantified. These proteins were the basis for further statistical analysis. The biggest difference of 88 significant altered proteins concerning their occurrence in perilymph was detected in all implanted (IGF-1 and AP) vs. all contralateral (IGF-1 and AP) ears. Whereas the number of significantly changed proteins in the comparison of the contralateral ears of IGF-1 and AP group amounts to nine, it is only three in the implanted ears (IGF-1 vs. AP). In order to cluster the quantified proteins, both all quantified proteins and the significantly varied ones per comparison were classified by Gene Ontology (GO) in the categories molecular function (Figure 6A) and biological process (Figure 6B). Since categories count nonexclusively, one protein may count in more than one category. The main part of all quantified proteins is involved in the molecular function binding, followed by catalytic activity and molecular function regulator. This distribution of the three most frequent categories was also found for the significantly altered protein occurrence in the comparison of implanted vs. contralateral and contralateral IGF-1 vs. contralateral AP. Regarding the classification of the biological process most proteins of all groups are involved in cellular process. All proteins and the altered proteins in the implanted vs. contralateral ears are the second most involved in biological regulation, followed by metabolic process. Proteins that are altered in the proteome of implanted IGF-1-treated ears in comparison to the AP-treated ones are only involved in cellular process, followed by immune system process.

To better understand the impact of IGF-1 delivery, the altered proteins in the comparison of the perilymph proteomes of the two implant conditions as well as the two contralateral conditions were further characterized concerning their name and their regulation. In the perilymph of IGF-1-treated ears the three altered proteins in comparison to the AP-treated ears were increased (Table 1).

The perilymph of the contralateral ears shows more variation in the comparison IGF-1- vs. AP-treated animals. Five proteins were increased and four proteins were decreased in the contralateral ears of the IGF-1 group (Table 2).

### 2.3. Histological Examination of the Inner Hair Cell’s Ribbon Synapses

**Figure 7:**
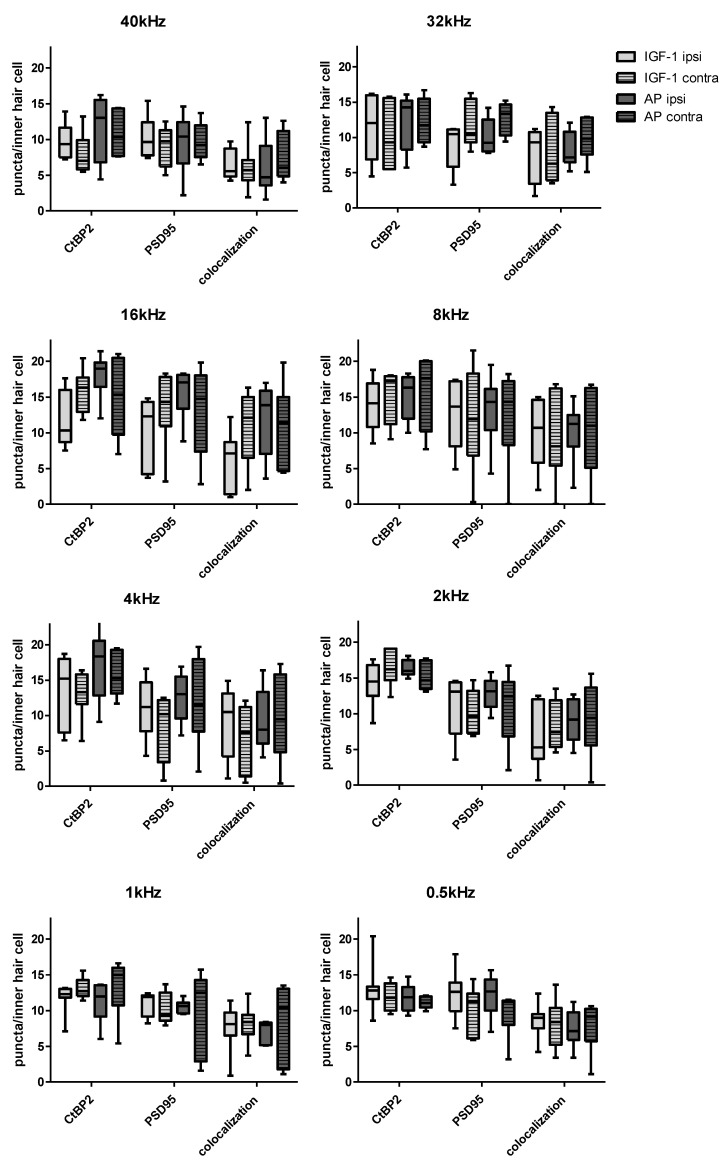
Synaptic counts per inner hair cell according to the corresponding frequency. Both CtBP2 and PSD95 puncta were counted individually. In addition, colocalized puncta in which the staining of CtBP2 and PSD95 overlapped were counted. Boxes display the mean and bars indicate the standard deviation.

All groups showed a variance in the ribbon number per inner hair cell (IHC) according to the corresponding frequency with the highest number in the middle-frequencies (Figure 7). This is already known from healthy individuals as well as noise exposed ones [44,46,47].

**Figure 8:**
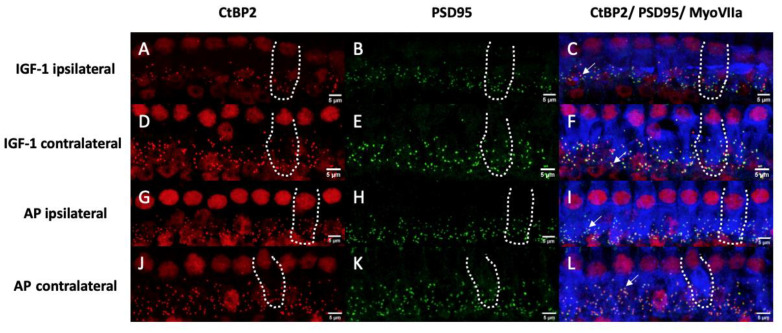
Representative images of the inner hair cells and their synapses corresponding to the 16 kHz region. Hair cells are labeled for myosin VIIa (blue), presynaptic ribbons for CtBP2 (red) and the postsynaptic density is labeled green. Yellow dots indicate an overlapping and therefore a colocalization of the CtBP2 and the PSD95 staining. Doted lines show the contour of a single IHC. Arrows point at synapses with colocalized staining. Left pictures (**A**,**D**,**G**,**J**) show only the CtBP2 staining and pictures in the middle only the PSD95 staining (**B**,**E**,**H**,**K**). The right pictures (**C**,**F**,**I**,**L**) display an overlay of all stainings. (**A**–**C**) belong to an implanted ear treated with IGF-1. (**D**–**F**) show a contralateral ear of an IGF-1 treated animal. (**G**–**I**) belong to an implanted ear treated with AP and (**J**–**L**) belong to a contralateral ear of an AP treated animal. Scale bars indicate 5 µm.

Figure 8A–L shows representative images of the 16 kHz region, that displays the highest variation in counts between all groups, from an implanted ear treated with IGF-1 (Figure 8A–C), a contralateral ear of an IGF-1-treated animal (Figure 8D–F), an implanted ear treated with AP (Figure 8G–I) and a contralateral ear of an animal treated with AP (Figure 8J–L).

Two-way ANOVA revealed no statistically significant difference between all four groups (IGF-1 ipsilateral, IGF-1 contralateral, AP ipsilateral, AP contralateral), but a tendency was apparent: In group comparison across all frequencies, AP ipsilateral had most frequently the highest absolute CtBP2 counts. The fewest CtBP2 puncta were counted most frequently in the IGF-1 ipsilateral group as well as the IGF-1 contralateral group.

It was similar when looking at the PSD95 counts. AP ipsilateral had most frequently the highest counts. The fewest PSD 95 counts were most frequently counted in the IGF-1 contralateral group. However, the most frequent highest colocalized counts were not seen in the AP ipsilateral but in the AP contralateral group.

The two IGF-1 groups had the fewest colocalized counts with equal frequency.

**Figure 9:**
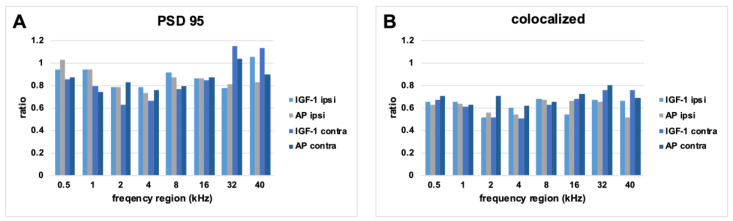
Ratio of mean values of PSD95 (**A**) and colocalized (**B**) points to CtBP2 counts for each condition. The mean CtBP2 counts for each condition was set as 1.

Since some noticeable differences in the absolute counts were seen between the groups, the ratio of the mean values of the PSD95 and colocalized points in relation to the mean number of CtBP2 puncta of the respective condition were also considered for further analysis (Figure 9). As a ration = 1 means that the same number of PSD95/colocalized puncta are present as CtBP2, this ratio can serve as a clue of which synapse parts have been more severely damaged in the remaining synapses. While the highest absolute PSD 95 counts were most frequent in the AP ipsilateral group, the highest ratio was most frequent in the IGF-1 ipsilateral group. A ratio above 1 was seen in five cases: AP ipsilateral 0.5 kHz, IGF-1 contralateral 32 kHz, AP contralateral 32 kHz, IGF-1 ipsilateral 40 kHz and IGF-1 contralateral 40 kHz. The IGF-1 contralateral group had most frequently the lowest ratio in both, PSD95 and colocalized counts. As with the absolute colocalized points, AP contralateral most often had the highest colocalization ratio.

## 3. Discussion

NIHL is a disorder with global importance [1], but unfortunately up to date there is no reliable treatment to regenerate the noise damaged ear. Since the mammalian inner hair cells are only partially able to regenerate [48], the prevention of damaged cochlear cells is preferable. Conventional hearing protection devices have limitations: the outcome depends on the user compliance, some (work) situations do not allow usage of, e.g., ear plugs and sometimes the timely occurrence of a noise insult is unpredictable. Therefore, pharmacological prevention may solve this problem, when a continuous delivery is given. The present study aimed to investigate the preventive effect of a long-term administration of IGF-1, being administered to the healthy ear, covering the time point of noise insult and lasting for one more week after trauma.

As already known, IGF-1 has antioxidant effects [31,32,33,49,50,51] and can promote the regeneration of cochlear ribbon synapses [35]. Its specific receptor is expressed not only in the immature but also in the mature inner ear and in newborn rats members of the IGF-family were found in the organ of corti, the modiolus and the stria vascularis [52]. After binding to its specific receptor (IGF1R), IGF-1 acts through various signaling pathways [51,53,54]. The three main pathways are PI3K/AKT, ERK/MAPK and stress kinases (p38 and JNK). In a murine cochlear explant culture, IGF-1 was able to protect hair cells against Neomycin ototoxicity through activation of both, PI3K/AKT and ERK/MAPK pathways [55,56]. These pathways are also involved in the pathogenesis of NIHL. This way blockade of the PI3K/AKT pathway due to silencing the PI3K regulatory subunit p85α or knockdown of Akt leads to an increased sensitivity against TTS in mice [57]. Interestingly, neither an alteration in the sensitivity to permanent threshold shift (PTS)-inducing noise nor in survival of outer hair cells (OHCs) was found [57]. Furthermore, the administration of activated protein C protects from NIHL by phosphorylation of AKT [58]. During the first 3–6 h after TTS-inducing noise, the MEK1/ ERK1/2/p90 RSK signaling pathway is activated and may serve as a protective mechanism [59]. This is supported by the fact that ERK2 knockout mice show a higher susceptibility to NIHL [60]. Another mechanism is the IGF-1 mediated modulation of glucose transport, which enhances neuronal survival during glucopenia [61]. Since Glucose is able to attenuate NIHL and reduce oxidative stress in hair cells [62], this mechanism may also play a key role.

As other studies have already shown that a local IGF-1 application to the round window membrane shortly before or directly after the noise trauma has a positive effect, we aimed to determine the effect of a long-term administration. The diffusion of IGF-1 through the membrane is challenging [38]. Therefore, a direct delivery in the scala tympani via osmotic pumps was chosen to apply the drug in a defined concentration, volume and duration. As Yamahara et al. showed a regenerative effect of 1 µg/mL on the ribbon synapses in vitro [35], we decided to fill the pumps with 2 µg/mL IGF-1. Although the amount of IGF-1 administered per hour is very low, it can be assumed that, without considering the unknown clearance, the delivered IGF-1 is enough to reach a perilymphatic concentration of 1 µg/mL within 24 h. Lin et al. also achieved a perilymphatic IGF-1 concentration of 1219 ng/mL 24 h after the treatment start by using ultrasound microbubbles, compared with the round window soaking method, which only reached a concentration of 731 ng/mL. To understand the results of our study more precisely it would be beneficial to measure the IGF-1 concentration in the perilymph. However, since the perilymph volume of guinea pigs is very small, the complete volume obtained was used for the proteome analysis. Therefore, we were not able to determine the IGF-1 concentration in the perilymph of the animals used in the study. However, in our view, it is quite reasonable to perform more detailed analyses of the pharmacokinetics of IGF-1 in the perilymph in subsequent studies. This knowledge may help to understand IGF-1 kinetics and effects in the cochlea and may subsequently lead to optimized IGF-1 inner ear therapy.

One week after starting the IGF-1 delivery, the surgery related threshold shifts of IGF-1 and AP implanted animals did not differ significantly (Figure 3). Nevertheless, the IGF-1 group showed slightly lower shifts in the lower frequencies, but not in the higher frequencies, where the highest surgery-related threshold shifts occurred. This is consistent with a study of Yamahara et al. who reported a smaller threshold shift in the low-frequency region of IGF-1 treated guinea pigs after cochlear implantation [34]. Therefore, both treatment groups had the same prerequisites when starting the noise trauma (Figure 3), a 4-h exposition to a modified musical piece. Other studies demonstrating beneficial effects of IGF-1 used 2 h white noise [36], 5 h 4 kHz octave band noise [37] or 5 h 8 kHz narrowband noise [38]. In the present study, the animals were exposed to 4 h of a sound file, that mimics the natural fluctuations of the temporal signal envelope and was adapted to the guinea pig hearing range. This should be considered when comparing the studies, as an effect of IGF-1 may occur not only depending on the frequency range but also on the intensity of the trauma. Interestingly, from the time point of the noise trauma, the effect of the IGF-1 changed, and the animals treated with IGF-1 showed higher threshold shifts at the implanted side, although both treatment groups started with the same prerequisites (Figure 4). Due to this negative tendency, no further animals were used in the interest of animal welfare, even though additional animals may provide a better indication of significance and may exclude the impact of random fluctuations. In the contralateral ears, no difference in hearing thresholds were seen between both treatment groups (Figure 5). Since IGF-1 has trophic effects to the inner ear [63], it may influence the healing of the round window membrane (RWM). Therefore, noise trauma may damage the sensory cells in the IGF-1-implanted ears more than in the AP-implanted ears. This hypothesis cannot be underlined with data since we do not have histological data on the RWM healing over time in this experiment. At the timepoint of tissue harvesting, i.e., 14 days after RWM incision, all RWM were visually inspected, and all were macroscopically closed. Additionally, IGF-1 treatment leads to an increased frequency of spontaneous excitatory postsynaptic currents in rat hippocampal neurons [64], which may explain the tendency towards a bigger noise damage.

In order to validate this tendency, histological evaluation of the inner hair cell synapses was performed. No significant differences were observed between the four groups (ipsilateral IGF-1, ipsilateral AP, contralateral IGF-1, contralateral AP) (Figure 7). All groups showed a reduction in the colocalized (paired) synapses [44,65] and the synaptic ribbons [66] in comparison to data of normal hearing guinea pigs. The normal hearing control ears in the study of Hickman et al. showed a broad peak of approximately 20 intact synapses/IHC in the frequency region between 8 and 16 kHz. This number slowly decreased < 15 towards the lower frequencies and more rapidly towards higher frequencies [44]. Whereas Song et al. reported that one week after a noise exposure of 4 h 95 dB SPL white noise for 7 consecutive days, guinea pigs showed the highest count at the PSD 95 per inner hair cell [65], our animals showed in accordance with other studies [67,68] the highest absolute count in the CtBP2 counts. Nevertheless, five conditions showed a CtBP2/PSD 95 ratio > 1 (Figure 9). As the normal ratio is one, this indicates damage of the presynaptic ribbons or increase in the postsynaptic density protein. The CtBP2/colocalization ratio roughly matches the published data of unpaired synapses [44]. Although the threshold shifts of implanted ears from day 7 suggest the strongest difference in the synapses at 8 kHz (Figure 4C), the largest difference in colocalized counts is seen at 16 kHz with AP-treated animals having higher counts (Figure 7). When comparing threshold shifts and synapse counts of implanted and contralateral ears, one should keep in mind that the threshold shift was set as the difference to day 0 pre noise. Therefore, there may be a surgery related side-difference. Overall, the IGF-1 treatment seems to negatively affect the absolute counts. Nevertheless, the ipsilateral ears of IGF-1-treated animals show most frequently the highest PSD95 ratio. However, it remains unclear if synapses with colocalized staining are functionally intact. This is supported by the fact that cochlear explants with regenerated synapses after kainate treatment showed an alteration in gene expression [69]. Gao et al. examined the effect of exogenous IGF-1 on the maintenance of ribbon synapses and determined a negative effect for concentrations ≥ 0.5 µg/mL, although there was no negative effect on hair cells or spiral ganglion neurons [70]. This is in contrast to Yamahara et al. who found a positive effect of 1 µg/mL on the synapse regeneration after excitotoxic trauma [35]. Both studies dealt with cochlear explants of different postnatal stages, but in the critical phase of the synapse formation [71]. Additionally, they used only the middle part of the organ of corti. However, in our study there is a tendency towards a better effect of IGF-1 treatment in the lower frequencies that are located more apical.

To understand the consequences of the IGF-1 application on the cochlear function, a proteome analysis of the perilymph was performed. Nowadays, human perilymph can be obtained from patients undergoing surgical procedures such as cochlear implantation [72], its analysis is a growing focus of research. This allows a better understanding of certain disease patterns [73] or mechanisms [74]. Despite human research, such studies are also increasingly performed in animal models. Therefore, the perilymph proteome of some laboratory animals including the guinea pig was defined [75,76]. We have identified 1478 proteins in the perilymph and quantified 466 proteins. Concerning these 466 proteins we are between the previous studies in terms of the detected proteins [75,76]. Additionally, the effect of noise on the perilymph metabolome [77,78] as well as the proteome [79,80] was investigated in previous studies. Consistent with those studies most of our quantified proteins are involved in the molecular function binding. Jongkamonwiwat et al. reported that 14 days after noise trauma, that cause temporary or permanent threshold shifts, proteins associated with protein synthesis are elevated, which indicates a recovery [79]. Since the biggest recovery of noise damaged ribbon synapses appears within the first week [44], we decided to use day 7 after noise insult, i.e., day 14 after infusion start, as endpoint. Interestingly, the change in the proteome towards the recovery phase seems to appear between day 7 and day 14 [79], indicating a faster structural than functional recovery. To clarify this one should compare proteome data of various time points after noise exposure and normal hearing animals in future studies. Delivery of IGF-1 led to three significantly altered proteins concerning their occurrence in the implanted ears and nine significantly altered proteins in the contralateral ears (Table 1 and Table 2). Although we expected the greatest differences between the implanted ears of both treatment groups, it may be that the implantation led to over coverage of hearing per se, as most of the changes are observed when comparing the implanted and contralateral ears.

Nevertheless, the IGF-1 treated animals showed higher levels of Collagen type VI alpha 3 (COL6A3) chain in both implanted and contralateral ears. Normally, various collagens are decreased in noise exposed mice [79,80]. As the collagens are also involved in the PI3K/AKT pathway [80,81], the increased COL6A3 may be directly upregulated by the IGF-1 treatment. In these perilymph samples additionally increased levels of Neural cell adhesion molecule 2 (NCAM2) were found. NCAMs are involved in neural development and neurite outgrowth in the inner ear [82] and have an impact on synaptic plasticity [83]. In the contralateral ears of the IGF-1-treated animals five proteins were increased and four proteins decreased. It is well known that contralateral spreading of drugs delivered to the cochlea is possible, e.g., through the cochlear aqueduct. One of the elevated proteins was Crystallin lambda 1, also known as glutamate dehydrogenase (GDH) [84]. Cochlear infusion of GDH causes a decrease in the compound action potential of the auditory nerve [85]. Metabolomic analysis of the noise exposed cochlear tissue revealed 25 up- and 15 down-regulated metabolites [86]. Among the upregulated metabolites were inosine-5’-monophasphate and pyridoxal 5-phosphate. In the contralateral ears of the IGF-1-treated animals the Inosine-5′-monophosphat dehydrogenase was decreased compared with the contralateral AP-treated ones. This enzyme is part of the guanine nucleotide biosynthesis and oxidizes inosine monophosphate. Therefore, a decrease may indicate that there was a lower nucleotide consumption during the noise exposure. On the other hand, it may lead to a reduced ROS formation due to diminished ATP being available at the mitochondria. Pyridoxal 5-phosphate is an important coenzyme of many enzymes involved in the amino acid metabolism and is synthetized by the pyridoxal kinase, which is increased in the perilymph of contralateral IGF-1-treated animals. This may indicate an increased repair process but can also indicate a higher need for repair. On the other hand, proteasome subunit beta is decreased, which is associated with proteolysis that occurs after noise exposure [79]. The reduced tyrosine 3-monooxygenase (also known as tyrosine hydroxylase) also suggests a positive effect of the IGF-1, since increased activity is associated with oxidative stress [87]. Furthermore, the increased D-dopachrome decarboxylase can be interpreted in two ways. First in a positive way, as it is downregulated in heart failures and activates ERK1/2 and Akt signaling [88]. On the other hand, it is increased in context of methamphetamine-induced neurotoxicity [89]. Vimentin is known to interact with IGF1R and promote axonal growth [90]. Our analysis showed a perilymphatic decrease in the contralateral ears of IGF-1-treated animals compared to AP treated. This may be explained by a higher IGF1R expression resulting in an increased uptake into the cells.

Considering all the above-mentioned points, there are both positive and negative effects of the IGF-1 application. As Gao et al. reported for the maintenance of ribbon synapses, there is a belly-shaped dose–response curve [70]. It can be assumed that the continuous administration of IGF-1 in the present study led to an accumulation in the cochlea and thus to an overdose. Although the exact clearance in the perilymph is not known, in human blood it is 8–16 h [91]. Therefore, even in patients with a primary IGF-1 deficiency, there is a need for dose adjustment during long-term treatment [92]. Nevertheless, previous studies reported both preventive and therapeutic effects in context of NIHL [36,37,38]. As the challenge of pharmacological prevention of NIHL in humans is the unknown timepoint of the noise insult and the accumulation of various noise insults, a continuous delivery seemed useful. The present study showed that there is need for adjustment of the application. Since it is possible to produce IGF-1 overexpressing stem cells [93], their circadian release may be one option to further investigate. Drug delivery via ROS-responsible nanoparticles represents another promising option [94]. They may avoid overdosage by a controlled release only in situations where the drug is needed. A systemic delivery does not seem to be useful due to the potential side effects, e.g., the involvement in neoplasia [95], especially for a preventive approach.

## 4. Materials and Methods

The experimental setup was previously published in detail by Malfeld et al. [43]. Therefore, published methods are only described briefly. Normal hearing guinea pigs were unilaterally implanted with a hook delivery-device (HDD) attached to an osmotic pump containing IGF-1 or artificial perilymph. One week after implantation (d 0) animals were exposed to a noise trauma of 4 h duration. The animals’ hearing status was determined on different timepoints using AABR measurements. On day 7, the animals were euthanized after a final AABR and apical perilymph sampling.

### 4.1. Pump Preparation

Osmotic minipumps (ALZET^®^ 2006, DURECT Corporation, Cupertino, CA, USA; pumping rate 0.15 µL/h) were filled and connected to a hook-delivery device (HDD) consisting of a commercially available silicone catheter (ALZET^®^ rat jugular catheter, DURECT Corporation, USA; 0.94 mm OD; 0.51 mm ID) and a small hook-shaped stainless-steel tip (Nordson Optimum^®^ #7018433, Nordson Deutschland GmbH, Erkrath, Germany) with an outer diameter of 0.31 mm [43]. Pumps for control animals were filled with artificial perilymph (AP; 145 mM NaCl, 2.7 mM KCl, 2.0 mM MgSO₄, 1.2 mM CaCl₂, 5.0 mM HEPES, pH = 7.4 with 0.1% guinea pig serum albumin) [96]. The pumps of the treatment group were filled with IGF-1 (Recombinant Human IGF-1, #100-11, PeproTech^®^ Germany, Hamburg, Germany) in a concentration of 2 μg/mL diluted in AP. The pumping rate of the osmotic pump results in a release of 0.3 ng IGF-1 per hour. During the priming time of 60 h each pumps’ function was macroscopic controlled by fluid drain out of the catheters’ tip [43]. After the in vivo experiment, the pumping of the explanted pumps was re-checked the same way.

### 4.2. Animals

Thirteen (seven in the IGF-1 group and six in the control group) male Dunkin Hartley guinea pigs (Charles River Laboratories, Châtillon, France) weighting between 350 g and 426 g were included in the study. Animals were housed in a temperature- and humidity-controlled room, exposed to a 24 h light-dark cycle (14 h/10 h) with free access to food and water. All experimental procedures were conducted in accordance with the German “Law on Protecting Animals” and with the European Communities Council Directive 2010/63/EU for the protection of animals used for experimental purposes. The use of animals for scientific purposes was permitted by the local authorities (Lower Saxony State Office for Consumer Protection and Food Safety (LAVES), Oldenburg, Germany, registration number 19/3145).

All procedures were performed under general anesthesia (intramuscular medetomidinhydrochloride 0.2 mg/kg, midazolam 1 mg/kg and fentanyl 0.025 mg/kg) with previous sedation (oral diazepam 4 mg/kg). During every anesthesia, the animals were placed on a heating pad. Areas to be incised were locally infiltrated with prilocaine. To reduce pain and to prevent infections the animals received subcutaneously 0.2 mg/kg meloxicam and 10 mg/kg enrofloxacin. Euthanasia was performed via intracardiac injection of not less than 300 mg/kg pentobarbital.

### 4.3. Acoustically Evoked Auditory Brainstem Response (AABR)—Measurement

Acoustic stimulation and recording of the auditory brainstem signals were performed using a Pilot Blankenfeld system that was modified for the use in guinea pigs (Pilot Blankenfeld Medizinisch—Elektronische Geräte GmbH, Blankenfelde-Mahlow, Germany) in a soundproof booth. Acoustic clicks were used to detect general auditory system thresholds. Frequency-specific acoustic thresholds were detected using tone bursts of 500 Hz, 1 kHz, 2 kHz, 4 kHz, 8 kHz, 16 kHz, 32 kHz and 40 kHz. Stimuli were presented by calibrated loudspeakers via a plastic cone placed in the outer ear canal. The contralateral ear was masked using white noise 30 dB lower than the stimulus. Determination of the hearing thresholds was done by visual inspection of AABR signals in the system’s analyze function at a maximum magnification of 700 nV/Div. The lowest stimulus intensity at which AABR signals could be detected was defined as the hearing threshold. Where it could not be defined up to the maximum sound stimulus level of 100 dB sound pressure level (SPL) peak (85 dB SPL peak for 40 kHz), the threshold was defined as 110 dB SPL peak or 95 dB SPL peak for 40 kHz. All animals fulfilled the criterion of initial normal hearing (click thresholds ≤ 40 dB SPL peak) [97].

The hearing threshold shift due to implantation was set as the difference between the measured thresholds of day −7 and day 0 pre noise exposure. The threshold shifts due to noise at different time points after exposure were set as the difference between the hearing thresholds of day 0 30 min post noise, day 1, and day 7 in comparison to the day 0 pre noise threshold.

### 4.4. Hook Delivery Device (HDD) Implantation

Following general anesthesia and additional local anesthesia of the areas to be incised, the scull was exposed. The left bulla was exposed using a retroauricular approach. A subcutaneous tunnel was built connecting the skin incision at the skull and the postauricular incision, in which the catheter was guided to the middle ear cavity. After opening the round window membrane using a micro-hook, the tip was inserted in the round window and the bulla was closed using UV cement (Tetric EvoFlow©, Ivoclar Vivadent, Schaan, Liechtenstein). The osmotic pump was placed in a subcutaneous pocket between the scapulae and the catheter placed on the head. To avoid tractive forces reaching the implant, a small, halved silicon tube was fixed at the skull guiding the catheter. The postauricular wound was closed in two layers and the wound at the skull was closed with u-sutures.

### 4.5. Noise Trauma

The anesthetized animal was placed in a sound-insulated box. Calibrated loudspeaker (DT48, 5 Ω, BeyerDynamic, Heilbronn, Germany) were bilaterally placed directly in front of the animal’s outer ear. Beethoven -5th Symphony, 4th movement: Allegro; Presto played by the Ensemble Reflector and recorded by PASCHENRecords was used as noise stimulus. The original audio was modified to generate a flat power spectrum between 200 Hz and 40 kHz (max. range 30 dB between frequencies), presented at a peak SPL of 120 dB. An exposure time of 4 h was chosen, which is known to cause a moderate temporary threshold shift in HDD implanted guinea pigs [43].

### 4.6. Perilymph Sampling and Analysis

14 days after starting the IGF-1 treatment, i.e., 7 days after TTS insult, animals were anaesthetized and following AABR measurement and µCT they received an additional local anesthetic nerve block. The bullae were exposed using a ventral approach. Perilymph was obtained by a cochleostomy in the apical region of the cochlea using the capillary forces of modified micro glass capillaries [72]. Each sample was transferred to an Eppendorf tube and stored at −80 °C until further analysis. Samples were processed based on an already described protocol [72]. In brief, they were mixed with Laemmli buffer, alkylated and separated by sodium dodecyl sulfate polyacrylamide gel electrophoresis (SDS-PAGE). Proteins were stained with Coomassie Brilliant Blue, and each lane was cut into three pieces which were further minimized into 1 mm³ pieces. These gel pieces were de-stained with two steps 100 µL 50% acetonitrile (ACN) and 20 mM ammonium bicarbonate (ABC), following dehydration with 100% ACN. Trypsin 10 ng/µL in 10% ACN/20 mM ABC) was added to the dried gel pieces and they were incubated overnight at 37 °C and gently shaking. Peptides were extracted with 50% ACN/5% trifluoracetic acid (TFA), followed by 50% ACN/0.5% TFA and 100% ACN. The peptide extracts were dried in a vacuum centrifuge and directly measured or stored dry at −20 °C. Redissolving was achieved using 2% ACN, 0.1% TFA with shaking at 800 rpm for 20 min. Following a centrifugation at 13,000× *g* the supernatant containing the peptides samples was analyzed with LC-MS using a DDA method with a RSLC and reversed phase chromatography and an Orbitrap Fusion Lumos (both Thermo Fisher, Waltham, MA, USA), as described recently [98]. Raw MS data were processed using Max Quant software [99] and Perseus software [100] and guinea pig entries of the Uniprot database [101]. Proteins were stated identified by a false discovery rate of 0.01 on protein and peptide level.

After normalization on median intensity values, the data were tested for significant differences between groups using student’s *t*-test. *p*-values < 0.05 were considered to be statistically significant. Proteins were mapped to the categories molecular function, biological process and cellular compartment by Gene Ontology (GO). GO offers the mapping of proteins at the UniProt Website http:/www.uniprot.org (accessed on 13 June 2022) based on the protein’s information in the UniProt Knowledgebase. Uncharacterized proteins were identified using Basic Local Alignment Search Tool (BLAST), which compares the identified protein sequence with sequences already included in a database. The perilymph data set is available publicly through this link: https://github.com/vianna-research/guinea-pig-perilymph-proteome-publication (accessed on 10 October 2022).

### 4.7. Tissue Preparation

After euthanasia, the bullae were harvested and the HDD’s position in the round window was checked. The HDD was explanted and the pumping of the osmotic pumps was rechecked. Both oval and round windows were opened and the cochlea was slowly perfused with 4% paraformaldehyde (PFA) via the round window followed by 1 h fixation in 4% PFA on ice. All following decalcification steps were performed with gentle agitation at room temperature (RT). After rinsing 3 × 10 min with phosphate-buffered saline (PBS), the specimens were decalcified in 10% ethylenediamine tetraacetic acid-disodium salt (EDTA, Sigma-Aldrich, Taufkirchen, Germany) in PBS, pH 7.4, with EDTA changes every 1–3 days. After 21 days in EDTA, the cochleae were washed 3 × 10 min with PBS. The basilar membrane was dissected in 8–9 pieces under a dissecting microscope and all pieces were transferred in PBS on glass slices to a humidity chamber for staining against the hair cells and the ribbon synapses. As the preparation was performed from apical to basal, the order of the pieces was noted. The staining protocol was modified from Wang et al. [102] and Shi et al. [67], based on our previously published protocol [103].

Permeabilization was achieved by 1% Triton-X (Sigma-Aldrich) in PBS for 1.5 h at room temperature. Subsequently the samples were blocked for not less than 4 h with blocking solution (5% normal horse serum (NHS)/1% Triton-X/PBS). The incubation time of 36 h at 4 °C for the primary antibodies (mouse anti PSD95, 1:200, Merck MAB1596; mouse anti CtBP2, 1:200, BD #612044; rabbit anti myosin VIIa 1:250, Novus Biol. NB 120-3481/5% NHS/PBS) was followed by 3 × 10 min washing steps with PBS at RT. Secondary antibodies (goat anti mouse IgG2a-Alexa488, 1:1000, Thermo Fisher #21131; goat anti mouse IgG1-Alexa 568, 1:1000, Thermo Fisher #21124; goat anti rabbit IgG-Cy5 1:500, Jackson #111-175-144/5% NHS/PBS) for 4 h at RT followed by three washing steps. Subsequently after removing the PBS, a drop of mounting medium (ProLong™ Gold Antifade Mountant, #P36930, Thermo Fisher) was added and the specimen was covered with a cover slip.

### 4.8. Synapse Quantification

Confocal Laser Scanning Microscopy (CLSM) of the cochlear whole mounts was performed using a Leica TCS SP8. To generate overview images of each fragment a 20-objective (HC PL APO CS2 20_/0.75 IMM, Fa. Leica) with immersion oil (Leica Microsystems #11513859) was used. Excitation was carried out using an argon-laser with 488 nm (Alexa 488), 568 nm (Alexa 568), and 650 nm (Cy5) emission maxima and the photomultiplier gates were adjusted to 504–572 nm, 582–644 nm, and 654–783 nm, respectively. Stacks were generated with a step size of 3 µm, resulting in a final size of 18 to 48 µm. The pinhole size was 56.7 µm and the zoom factor was 0.75. Frequency-mapping of each cochlea was conducted using a custom-made ImageJ plugin (https://www.masseyeandear.org/research/otolaryngology/eaton-peabody-laboratories/histology-core, accessed on 17 March 2021). Afterwards, detailed images of the inner hair cells at regions corresponding to all tested frequencies via AABR measurements were created. For this purpose, a 63-objective (HC CL APO CS2 63_/1.40 OIL, Fa. Leica) with immersion oil was used and an additional optical zoom of about 1.5 (1.25 to 1.8) was added. The z-stacks with a final size of 6.5 to 19.5 µm were generated in 0.5 µm steps with a pinhole size of 95.5 µm. To investigate the condition of the inner ear ribbon synapses, the presynaptic (CtBP2), postsynaptic (PSD95) and colocalized puncta, which indicate an intact synapse [46,104], were counted manually in each frequency region corresponding to AABR measurements (0.5, 1, 2, 4, 8, 16, 32, 40 kHz) containing 6-10 inner hair cells (IHCs) using ImageJ. When the hair cells in a region were destroyed during the dissection, the synapses were counted, if possible, in 6–10 IHCs in the area up to a quarter of the distance to the next frequency.

### 4.9. Statistical Analysis

The person who conducted the animal experiments was blinded for the pump filling, i.e., the experimental group, until the animal’s death. The person who performed the imaging and counting of the synapses was blinded as well.

The statistical analysis of ABR and histological data was performed using GraphPad Prism© version 8.4.3. Data were checked for normal distribution using the Kolmogorov–Smirnov test. Hearing threshold or threshold shifts at different time points between groups were analyzed using an unpaired t-test. Synapse counts were compared across all groups using a 2-way ANOVA. The data are reported as mean ± standard deviation (SD). Statistical significance was considered and depicted at *p* ≤ 0.05 (*) and *p* ≤ 0.01 (**). For information about the statistical analysis of the perilymph data see Section 4.6.

## 5. Conclusions

Contrary to the literature, the present study did not show any significant improvement in NIHL with preventive administration of IGF-1 via osmotic pumps. There were indications of increased sensitivity to noise after IGF-1 application. Nevertheless, changes in the perilymph proteome were also shown, which could indicate reduced damage and better repair mechanisms. Future studies using IGF-1 should consider investigating other continuous delivery methods for prevention of NIHL but should avoid overdosage.

## Figures and Tables

**Table 1:** Significant (*p* < 0.05) altered proteins in the ears implanted with IGF-1 in comparison to the ones implanted with AP. They are all increased (↑) in the IGF-1 treatment group.

UniProt ID	Protein Name	↑/↓	*p*-Value	Ratio (Logarithm to Base 2)
H0VIY0	Collagen type VI alpha 3 chain	↑	0.029	2.003
H0VRB7	Ig-like domain-containing protein	↑	0.022	2.282
H0UXT4	Neural cell adhesion molecule 2	↑	0.002	2.654

**Table 2:** Significant (*p* < 0.05) increased (↑)/decreased (↓) proteins in the contralateral ears of the animals implanted with IGF-1 in comparison to the contralateral ears of the ones implanted with AP. UniProt IDs marked with * were linked to an uncharacterized protein and the identification of the name was done using Basic Local Alignment Search Tool (BLAST).

UniProt ID	Protein Name	↑/↓	*p*-Value	Ratio (Logarithm to Base 2)
H0VIY0	Collagen type VI alpha 3 chain	↑	0.003	1.768
H0V0H2	Crystallin lambda 1	↑	0.01	1.549
A0A286XVA7	Proteasome subunit beta	↓	0.012	−1.021
H0VFS3	Inosine-5’-monophosphate dehydrogenase	↓	0.008	−2.219
A0A286Y4U7 *	D-dopachrome decarboxylase	↑	0.034	1.422
A0A286Y3H7	Pyridoxal kinase	↑	0.007	1.301
A0A286XP66	Tyrosine 3-monooxygenase/tryptophan 5-monooxygenase activation protein gamma	↓	0.016	−0.843
H0UVC5	Vimentin	↓	0.022	−0.809
H0UT45 *	Alpha-2-macroglobulin	↑	0.044	0.851

## Data Availability

The data presented in this study are available upon request from the corresponding author. The perilymph data set is available publicly through this link: https://github.com/vianna-research/guinea-pig-perilymph-proteome-publication (accessed on 10 October 2022).
